# A retrospective comparative study of cyanoacrylate embolization and radiofrequency ablation in the treatment of incompetent perforator veins

**DOI:** 10.1097/MD.0000000000043070

**Published:** 2025-06-27

**Authors:** Oguz Arslanturk, Sitki Akin Turan, Ali Kemal Gur

**Affiliations:** a Department of Cardiovascular Surgery, Zonguldak Bülent Ecevit University, Faculty of Medicine, Zonguldak, Turkey.

**Keywords:** chronic venous insufficiency, cyanoacrylate, perforator vein, radiofrequency, ulcer healing, vein closure

## Abstract

Chronic venous insufficiency (CVI) is a progressive disease that is often associated with incompetent perforator veins (IPVs) that exacerbate venous hypertension, ulceration, and reduced quality of life. Minimally invasive techniques such as cyanoacrylate ablation (CA) and radiofrequency ablation (RFA) have become alternatives to traditional surgical methods. However, comparative data on their efficacy and safety remain scarce. We retrospectively analyzed 118 patients with IPVs who underwent either CA (n = 55) or RFA (n = 63) between January 2018 and January 2024. Outcomes included vein closure rates at 1, 3, and 12 months; ulcer healing rates; quality of life (CIVIQ-20 scores); and procedure-related complications. At 12 months, the RFA group demonstrated a significantly higher full closure rate (88.8%) than the CA group (72.7%; *P* = .044). Ulcer healing rates were similar between groups (85% vs 80%; *P* = .134), while CIVIQ-20 scores improved significantly in the RFA group (−19.5 ± 4.5 vs −15.5 ± 3.5; *P* = .041). Both techniques exhibited low complication rates with no significant differences in adverse events. CA and RFA are effective and safe options for treating IPVs in patients with advanced CVI. However, RFA demonstrates superior long-term vein closure rates and greater improvements in quality of life, making it the preferred approach in many cases. While CA remains a viable alternative for selected patients, these findings underscore the significance of tailoring treatment strategies to optimize patient outcomes, particularly favoring RFA for its long-term efficacy.

## 1. Introduction

Chronic venous insufficiency (CVI) is a progressive vascular disorder characterized by an impaired venous return. It is associated with complications such as edema, venous ulceration, and a significant decline in the quality of life, representing a widespread global health concern.^[1,2]^ Untreated or persistent superficial varicose veins lead to advanced CVI, including lower extremity swelling, eczema, pigmentation, bleeding, and ulceration; the most common cause is perforator vein insufficiency.^[3,4]^ In recent years, the treatment of perforating vein insufficiency has evolved into using various minimally invasive techniques, such as ultrasound-guided endovascular thermal ablation or sclerotherapy.^[5,6]^ 2022 guidelines from the European Society for Vascular Surgery (ESVS) suggest that endovenous ablation, division, or ligation should be considered for managing incompetent perforating veins (IPVs) in patients with chronic venous disease, with a recommendation grade of Class IIa and evidence-level C.^[7]^ These recommendations are in line with an increasing preference for minimally invasive approaches. This is particularly important in cases where fragile skin around venous ulcers increases the risk of surgical complications. While traditional surgical methods such as the Linton procedure and subfascial endoscopic perforator surgery have demonstrated efficacy, advancements in minimally invasive techniques offer promising results in managing IPVs, with reduced complications and faster recovery.^[8,9]^ Minimally invasive techniques such as cyanoacrylate ablation (CA) and radiofrequency ablation (RFA) are effective alternatives for treating IPVs.^[10,11]^ These methods offer the advantages of reduced procedural trauma, faster recovery, and improved patient satisfaction compared with traditional surgical approaches. Despite the increasing adoption, comparative evidence regarding the safety, efficacy, and long-term outcomes of CA and RFA in treating IPVs remains limited. This study aimed to address this gap by evaluating and comparing the clinical success, safety profiles, and patient-reported outcomes of these 2 techniques to provide insights into optimizing treatment strategies for this challenging condition

## 2. Materials and methods

This retrospective study evaluated patients with CVI and IPVs who underwent CA or RFA between January 2018 and January 2024. Inclusion criteria encompassed patients classified as CEAP C6 with active venous ulcers, confirmed perforator vein incompetence on duplex ultrasound (diameter ≥ 3.5 mm and reflux duration > 0.5 seconds), and a history of at least 3 months of failed conservative treatment, including compression therapy. The exclusion criteria included active deep vein thrombosis (DVT) or pulmonary embolism within 6 months, pregnancy or breastfeeding, prior intervention on the same IPVs, known hypersensitivity to cyanoacrylate adhesive or procedural materials, inability to comply with follow-up, ongoing anticoagulant therapy that could not be interrupted, and severe comorbidities or terminal illness with a life expectancy of 1 year. These criteria ensured a well-defined study population with advanced venous disease while minimizing confounding factors. This retrospective study was conducted in accordance with the principles of the Declaration of Helsinki. Institutional approval was granted by the Ethics Committee of Zonguldak Bülent Ecevit University (approval no. 2024/01). Due to the retrospective nature of this study, the requirement for individual patient informed consent was waived by the ethics committee, as the study involved analysis of existing clinical data with no additional interventions or patient contact. Patient confidentiality was maintained throughout the data collection and analysis process, with all patient identifiers removed from the study dataset.

### 2.1. Operative techniques

Patients underwent duplex ultrasonography to identify the IPVs (diameter > 3.5 mm and reflux duration > 0.5 seconds). Patients were assigned to either the CA or RFA group based on clinical evaluation and physician discretion.

#### 2.1.1. Cyanoacrylate embolization

After appropriate sterilization and draping, the patient was placed in the reverse Trendelenburg position. Duplex ultrasonography was performed using a Mindray DP-10 portable duplex scanner (Guangdong, China) to identify target perforator veins in transverse and longitudinal views. For local anesthesia, approximately 0.5 mL of 1% lidocaine was administered at each procedural site. The embolization procedure used 21-gauge needles and 2 mL syringes prefilled with 0.5 mL cyanoacrylate adhesive (VenaBlock Venous Closure System; Invamed, Ankara, Turkey). Under ultrasonographic guidance, a needle was inserted longitudinally into the midpoint of the IPVs. After confirming the correct positioning of the needle, cyanoacrylate was rapidly injected to avoid premature polymerization at the needle tip. The injection was monitored in real-time using duplex ultrasonography, and gentle compression was applied to the injection site for approximately 10 seconds to promote adhesion. This procedure was repeated for each IPVs target. Following the removal of the needle, a multilayer compression dressing (3 or 4 layers) was applied to the treated leg to finalize the procedure.

#### 2.1.2. Radiofrequency ablation

The patient was placed in the reverse Trendelenburg position on an electronically adjustable table, and duplex ultrasonography was performed using a Mindray DP-10 portable duplex scanner (Guangdong, China). The previously identified IPVs were confirmed and marked by an operating surgeon. Legs were prepared under sterile conditions. Utilizing a 12 MHz ultrasound transducer encased in a sterile sheath, perforating vessels above the ulcer were selected for treatment. For vessels with greater flexibility, a stylet from the ClosureFast radiofrequency (RFS) catheter (Medtronic, Minneapolis) was used for percutaneous access. For stiff or calcified vessels, a No. 11 scalpel was used to create an entry point for the catheter insertion. Under ultrasonography guidance, the stylet was advanced at a 45° angle, and the transducer was rotated 90° to confirm accurate positioning in 2 orthogonal planes. The RFS catheter was placed at the junction of the perforator vein and fascia and then retracted 2 to 3 mm from the deep vein connection. After confirming the position of the catheter, tumescent anesthesia was administered by injecting lidocaine along the catheter track to the level of the fascia using a 25-gauge needle. The patient was repositioned in the Trendelenburg position and catheter placement was confirmed. Radiofrequency energy was applied, treating each quadrant of the vein for 1 minute while maintaining an impedance below 400 Ω and a target temperature of 85°C. The catheter was retracted incrementally by 3 to 5 mm, and this process was repeated until the entire perforator vein length was treated in the 4 quadrants. Upon the completion of the procedure, the catheter was removed, and a 3- or 4-layer compression dressing was applied to the treated leg

### 2.2. Outcome measurements

The primary outcome was the closure of IPVs at 1, 3, and 12 months postprocedure, as confirmed by duplex ultrasound. The closure was categorized as fully closed, partially closed, or open.

Secondary outcomes included changes in ulcer healing rates and size, quality of life improvements measured with the Chronic Venous Insufficiency Quality of Life Questionnaire-20 (CIVIQ-20), and procedure-related complications such as paresthesia, skin burns, thrombophlebitis, and DVT documented during follow-up visits.

These outcomes offer a comprehensive evaluation of the clinical efficacy, safety, and overall impact of the procedures on patients’ quality of life.

### 2.3. Statistical analysis

Statistical analyses were performed using SPSS (version 27.0; SPSS Inc., Chicago). The Kolmogorov–Smirnov test was used to assess the normality of the quantitative variables. Normally distributed variables were compared using independent sample t-tests, whereas non-normally distributed variables were analyzed using the Mann–Whitney *U* test. A chi-square analysis was conducted to evaluate the relationships between qualitative variables. Normally distributed quantitative data were expressed as mean ± standard deviation, whereas qualitative data were reported as frequencies and percentages. Adjustments for potential confounding variables were not performed because the limited sample size restricted the ability to conduct multivariable analyses. Statistical significance was set at *P* < .05 significant.

## 3. Results

The study included 118 patients with IPVs; 55 were treated with CA, and 63 with RFA. The baseline demographics and clinical characteristics were comparable between the groups (Table 1). There were no significant differences in sex distribution, age, body mass index (BMI), or CEAP classification (C5/C6).

**Table 1:** Patient demographics and clinical characteristics.

Variables	CEA group (n = 55)	RFA group (n = 63)	*P*-value*
Gender, male	22 (40%)	28 (44.4%)	.764
CEAP class C5/C6 (n)	27/28	31/32	NA
Diabetes mellitus	14 (25.4%)	18 (28.5%)	.863
Deep vein reflux	17 (30.9%)	21 (33.3%)	.933

	Mean ± sd		*P*-value†
BMI (kg/m²)	35.2 ± 9.8	34.9 ± 10.2	.842
Age (yr)	62.6 ± 12.6	61.8 ± 13.0	.721

BMI = body mass, index, CEA = cyanoacrylate embolization, CEAP = Clinical-Etiology-Anatomy-Pathophysiology Class, RFA = radiofrequency ablation.

* Chi square test.

† Indipendent *t* test.

### 3.1. Primary outcome: vein closure rates

**Figure 1.**
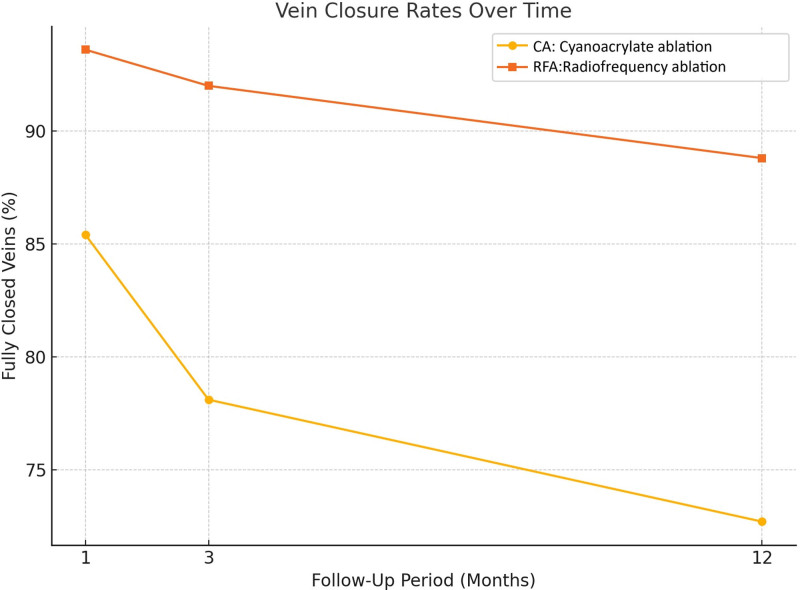
Vein closure rates over time. Comparison of fully closed vein percentages between CA and RFA at 1, 3, and 12 mo follow-up. RFA consistently demonstrated higher closure rates compared to CEA, with statistically significant differences observed at 12 mo (*P* < .05). CA = cyanoacrylate ablation, CEA = cyanoacrylate embolization, RFA = radiofrequency ablation.

Vein closure success rates varied significantly between the 2 techniques during the 12-month follow-up period. At 1 month, complete closure was achieved in 85.4% of the patients in the CA group and 93.6% in the RFA group, although the difference was not statistically significant (*P* = .244). By 3 months, the closure rates had increased to 92.0% in the RFA group and 78.1% in the CA group, with a statistically significant difference (*P* = .064). At 12 months, the RFA group demonstrated significantly higher efficacy, achieving a full closure rate of 88.8% %compared to 72.7% in the CA group (*P* = .044) (Fig. 1) (Table 2).

**Table 2:** Comparison of vein closure outcomes between cyanoacrylate embolization and radiofrequency ablation at 1, 3, and 12 mo follow-up.

Follow-up period	Outcome	CEA (n, %) (n = 55)	RFA (n, %) (n = 63)	*P*-value
1 mo	Fully closed	47 (85.4%)	59 (93.6%)	.244
	Partially closed	6 (10.9%)	2 (3.1%)	
	Open	2 (3.6%)	1 (1.5%)	
3 mo	Fully closed	43 (78.1%)	58 (92.0%)	.064
	Partially closed	9 (16.3%)	3 (5.4%)	
	Open	3 (5.4%)	2 (3.1%)	
12 mo	Fully closed	40 (72.7%)	56 (88.8%)	.044*
	Partially closed	11 (20.0%)	4 (6.3%)	
	Open	4 (7.2%)	3 (4.7%)	

CEA = cyanoacrylate embolization, RFA = radiofrequency ablation.

* *P* < .05, statistically significant value.

### 3.2. Secondary outcomes: ulcer healing and quality of life

Among the patients with active ulcers, both treatment approaches achieved notable healing rates at the 12-month follow-up. Ulcer healing was observed in 85% and 80% of the patients in the CA and RFA groups, respectively, with no statistically significant differences (*P* = .134). Similarly, reductions in ulcer size were reported in 75% of patients undergoing CA and 70% of those undergoing RFA (*P* = .152). In contrast, quality of life improvements, evaluated using the CIVIQ-20 questionnaire, were significantly greater in the RFA group (−19.5 ± 4.5) compared to the CA group (−15.5 ± 3.5) (*P* = .041) (Table 3).

**Table 3:** Comparison of clinical and quality of life outcomes between cyanoacrylate embolization and radiofrequency ablation.

Outcome	CEA n = 28	RFA n = 32	*P*-value†
Patients with ulcer healing	24 (85%)	25 (80%)	.134
Reduction in ulcer size (mm)	15 (75%)	14 (70%)	.152

	Mean ± sd		*P*-value‡
CIVIQ-20 score reduction (points, 12 mo)	−15.5 ± 3.5	−19.5 ± 4.5	.041*

CEA = cyanoacrylate embolization, CIVIQ-20 = The Chronic Venous Insuficiency Quality of life Questionnaire, RFA = radiofrequency ablation.

† Chi square test.

‡ Indipendent *t* test.

* *P* < .05, statistically significant value.

### 3.3. Procedure-related complications

**Figure 2.**
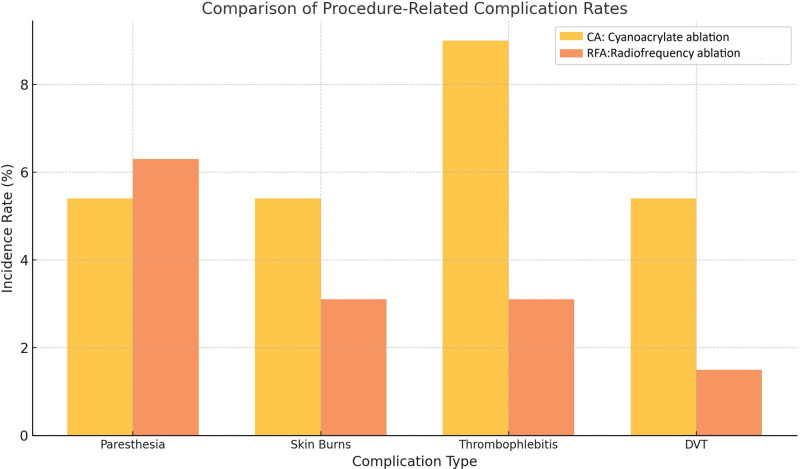
Comparison of procedure-related complication rates. Bar chart comparing the incidence rates of procedure-related complications between CA and RFA. Complications analyzed include paresthesia, skin burns, thrombophlebitis, and DVT. Both techniques exhibited low complication rates, with no statistically significant differences observed between the groups (*P* > .05). CA = cyanoacrylate ablation, DVT = deep vein thrombosis, RFA = radiofrequency ablation.

Adverse events were uncommon and were predominantly mild in both treatment groups (Table 4). The paresthesia rates were comparable between the CA and RFA groups (5.4% vs 6.3%, *P* = .960). Instances of skin burns and thrombophlebitis were rare, and no significant differences were observed. DVT occurred in 5.4% of the patients treated with CA and 1.5% of those treated with RFA; however, this difference was not statistically significant (*P* = .517) (Fig. 2).

**Table 4:** Procedure-related complications in patients undergoing cyanoacrylate embolization versus radiofrequency ablation.

Complication	CEA n = 55	RFA n = 62	*P*-value
Paresthesia	3 (5.4%)	4 (6.3%)	.960
Skin burns	3 (5.4%)	2 (3.1%)	.877
Thrombophlebitis	5 (9.0%)	2 (3.1%)	.334
Deep vein trombosis	3 (5.4%)	1 (1.5%)	.517

CEA = cyanoacrylate embolization, RFA = radiofrequency ablation.

† Chi square test.

## 4. Discussion

IPVs have been demonstrated as the cause of persistent or recurrent varicose veins.^[12,13]^ Minimally invasive approaches to IPVs treatment have shown enhanced healing of ulcerations and reduced recurrence in patients with advanced venous diseases.^[5,12]^ IPVs are critical contributors to the progression and recurrence of lower-limb venous insufficiency; however, there is no clear consensus on the optimal treatment approach.^[6,14,15]^ This study offers important perspectives on the relative effectiveness and safety of CA and RFA in managing IPVs in individuals with advanced CVI. Our results demonstrate that while both techniques effectively promote vein closure and improve clinical outcomes, RFA offers superior long-term vein closure rates, which is particularly evident at the 12-month follow-up. This highlights its potential as a reliable option for achieving sustained therapeutic outcomes in patients.

In a comparative study of RFA, laser ablation, and foam sclerotherapy for perforator vein closure, Hager et al identified RFA as the most reliable method, highlighted morbid obesity as a significant predictor of failure across all modalities, and demonstrated the efficacy of thermal ablation as a secondary treatment after foam sclerotherapy failure.^[10]^ In our study, the 12-month full closure rate with RFA (88.8%) was higher than that with CA (72.7%; *P* = .044), suggesting that RFA may provide greater durability in veins closure. This aligns with prior research such as that of Hager et al, who identified RFA as a highly effective modality for IPVs treatment, particularly for achieving consistent vein closure over time. On the other hand, Mordhorst et al reported that CA for treating IPVs achieved a closure rate of 86.5% at 72 ± 9 days, with no DVT and only 1 case of septic thrombophlebitis successfully managed with antibiotics.^[11]^ While the initial closure rates for CA were promising (85.4% at 1 month), the decline in efficacy over time underscores the need for further optimization of this technique to enhance long-term outcomes. Similarly, Mordhorst et al observed high initial closure rates with cyanoacrylate; however, the durability of the results varied, necessitating careful patient selection and follow-up.

Kiguchi et al demonstrated that ultrasound-guided sclerotherapy-induced thrombosis of IPVs markedly enhanced venous ulcer healing and identified complete IPVs closure as the strongest predictor of healing; in contrast, male gender and warfarin use were associated with reduced thrombosis rates.^[12]^ These findings further emphasize the importance of achieving complete IPVs closure as the primary therapeutic goal in patients with venous ulceration. The combined approach of CA and sclerotherapy proved to be a feasible and effective outpatient treatment modality for recurrent lower extremity venous insufficiency caused by inadequate perforators, achieving high occlusion rates and ulcer healing in their study by Prasad et al.^[6]^ However, deep venous dilatation of the cyanoacrylate occurred in 4.8% of patients. Significant long-term thrombophlebitis was observed in 38.5% of limbs, emphasizing the need for careful patient selection and follow-up. The SeCure trial demonstrated that endovenous laser ablation with a 400 μm optical fiber and 1470 nm laser is a safe and effective treatment for pathologic perforator veins in advanced venous disease, achieving significant ulcer healing and quality of life improvements with minimal adverse events.^[16]^ In our study, both CA and RFA demonstrated high ulcer healing rates at 12 months, with similar differences between the groups (85% vs 80%; *P* = .134). This finding reinforces the efficacy of both techniques in addressing the clinical manifestations of CVI, including ulceration. However, the greater improvement in CIVIQ-20 scores observed in the RFA group (−19.5 ± 4.5) compared to the CA group (−15.5 ± 3.5; *P* = .041) suggests that RFA may offer superior enhancements in patient-reported quality of life. These findings are consistent with those of previous studies highlighting the benefits of thermal ablation techniques in improving functional outcomes and patient satisfaction.

In an ultrasound-guided cyanoacrylate adhesive perforator embolization (CAPE) study, an occlusion rate of 76 % was achieved at 3 months for IPVs with no serious complications reported.^[17]^ In another study on cyanoacrylate treatment for perforator vein closure in CEAP-6 patients, 87.1% achieved successful closure at 12 months with a significant reduction in ulcer size and complete healing.^[18]^ The present study contributes to this body of evidence by directly comparing CA and RFA and offering insights into their relative strengths and limitations.

The retrospective design is the most important limitation of the study. Second, the follow-up period was limited to 12 months; a longer follow-up might provide additional insights into the long-term durability of both techniques. Finally, the number of patients included in the study was a significant limiting factor due to the rarity of these cases. However, we took several measures to strengthen the validity of our findings. The demographic and clinicopathological characteristics of our patients were comparable between both groups, as demonstrated in Table 1. We implemented strict inclusion criteria with standardized venous insufficiency parameters across all patients. The research was conducted by a cardiovascular surgery team with at least 5 years of experience, and both techniques were performed by physicians with equivalent expertise, ensuring procedural consistency. Throughout the 12-month follow-up period, we conducted systematic evaluations at predefined intervals (1, 3, and 12 months), allowing for consistent and objective assessment of outcomes.

In conclusion, both CA and RFA are effective and safe modalities for treating IPVs in patients with advanced CVI. Both techniques didn’t show statistically superiority over another for treatment purposes. While RFA demonstrates advantages in long-term vein closure rates and quality of life improvements, CA remains a valuable alternative with comparable safety and ulcer healing outcomes. These findings support the continued adoption of minimally invasive techniques for managing advanced venous disease while emphasizing the importance of individualized treatment selection. Factors such as patient preferences, anatomical considerations, comorbidities, and specific clinical manifestations should guide the choice between these viable options to optimize outcomes for each patient. 

## Author contributions

**Conceptualization:** Oguz Arslanturk, Sitki Akin Turan, Ali Kemal Gur.

**Data curation:** Oguz Arslanturk, Sitki Akin Turan.

**Formal analysis:** Oguz Arslanturk, Sitki Akin Turan, Ali Kemal Gur.

**Investigation:** Oguz Arslanturk, Sitki Akin Turan.

**Methodology:** Oguz Arslanturk, Sitki Akin Turan, Ali Kemal Gur.

**Project administration:** Oguz Arslanturk.

**Resources:** Sitki Akin Turan, Ali Kemal Gur.

**Software:** Sitki Akin Turan, Ali Kemal Gur.

**Supervision:** Oguz Arslanturk.

**Validation:** Oguz Arslanturk, Sitki Akin Turan, Ali Kemal Gur.

**Visualization:** Oguz Arslanturk, Sitki Akin Turan.

**Writing – original draft:** Oguz Arslanturk, Sitki Akin Turan, Ali Kemal Gur.

**Writing – review & editing:** Oguz Arslanturk, Sitki Akin Turan.
